# Acute fulminant pseudomembranous colitis which developed after ileostomy closure and required emergent total colectomy: a case report

**DOI:** 10.1186/1752-1947-6-130

**Published:** 2012-05-14

**Authors:** Iku Abe, Yutaka J Kawamura, Junichi Sasaki, Fumio Konishi

**Affiliations:** 1 Department of Surgery, Jichi Medical University Saitama Medical Center, 1-847, Amanuma-cho, Saitama-shi, Saitama, Omiya-ku, 330-8503, Japan

## Abstract

**Introduction:**

Pseudomembranous colitis is known to be caused by *Clostridium difficile;* and, in 3% to 8% of patients, it lapses into an aggressive clinical course that is described as fulminant. We present here a case of extremely rapid and fatal fulminant pseudomembranous colitis that developed after ileostomy closure, a minor surgical procedure. To the best of our knowledge, this is the first case report of fatal fulminant pseudomembranous colitis after closure of a diversion ileostomy in an adult.

**Case presentation:**

A 69-year-old Japanese man, who had previously undergone low anterior resection and creation of a diverting ileostomy for stage III rectal carcinoma was admitted for ileostomy closure. Preoperatively, he received oral kanamycin and metronidazole along with parenteral cefmetazole. His surgery and postoperative course were uneventful until the third postoperative day, when fever and watery diarrhea became apparent. The next day he presented with epigastric and left lower abdominal pain. Computed tomography revealed a slightly distended colon. Later that night, his blood pressure fell and intravenous infusion was started. In the early morning of the fifth postoperative day, his blood pressure could be maintained only with a vasopressor. Follow-up computed tomography demonstrated severe colonic dilation. A colonoscopy confirmed the presence of pseudomembranous colitis, and so oral vancomycin was administered immediately. However, within three hours of the administration, his condition rapidly deteriorated into shock. Although an emergent total colectomy with creation of an end ileostomy was performed, our patient died 26 hours after the surgery. The histopathological examination was consistent with pseudomembranous colitis.

**Conclusion:**

It is important to recognize that, although rare, there is a type of extremely aggressive pseudomembranous colitis in which the usual waiting period for medical treatment might be lethal. We consider that colonoscopy and computed tomography are helpful to decide the necessity of emergent surgical treatment without delay.

## Introduction

Pseudomembranous colitis is caused by an infection with the bacterium *Clostridium difficile* (CDI) [1]. It is well known that in some patients with CDI, the ailment lapses into a fulminant state. For these patients, the initial treatment of choice is nonsurgical; and then, if such treatment proves to be ineffective, a surgical consult is considered [2]. We present a case of fatal fulminant pseudomembranous colitis showing extremely rapid progress after a minor surgical procedure. To the best of our knowledge, this case is the first reported case of fulminant pseudomembranous colitis after ileostomy closure in the English literature. We emphasize the importance of more prompt decision making and intervention than those described in the conventional treatment algorithm for CDI.

## Case presentation

A 69-year-old Japanese man was admitted to our hospital for elective ileostomy closure. Ten months earlier, after chemoradiation therapy, he had undergone a low anterior resection and creation of a diverting ileostomy for rectal carcinoma, which was complicated with postoperative leakage. Histopathological study revealed stage III rectal carcinoma (T3N1M0). He had a past medical history of hypertension, cerebral hemorrhage and gastric ulcer and was taking a proton-pump inhibitor.

On the day before the elective surgery, he received oral kanamycin and metronidazole as a chemical preparation. Additionally, immediately before the surgery, one dose of parenteral cefmetazole was given. Ileostomy closure was completed in 31 minutes without any intraoperative complications.

His postoperative course was uneventful until the third postoperative day, when his body temperature rose to 37.8°C. His bowel movement became frequent at 15 times a day. However, because he had no other signs or symptoms suggestive of infection, and because frequent bowel movement is not uncommon after low anterior resections, especially after those complicated with postoperative leakage, careful observation was continued without further examination or additional treatment.

**Figure 1:**
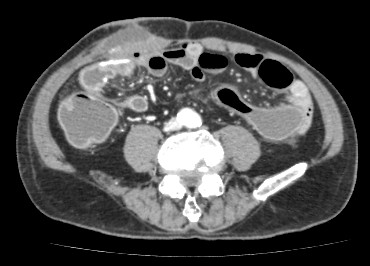
**Abdominal computed tomography on the third postoperative day. **A CT scan demonstrated that subcutaneous fluid had collected adjacent to the surgical wound and that the colon had become slightly distended and showed no wall thickening.

On the fourth postoperative day, in addition to the aforementioned symptoms, he presented with epigastric and left lower abdominal pain. A computed tomography (CT) scan (Figure 1) revealed that subcutaneous fluid had collected adjacent to the surgical wound. CT also demonstrated a slightly distended colon without wall thickening. We immediately opened the surgical wound and drained the fluid, which was sanguineous. After the drainage, his body temperature returned to within the normal range (36.7°C). That night, at 10:30 p.m., his blood pressure fell to 77/59mmHg and intravenous infusion was started.

**Figure 2:**
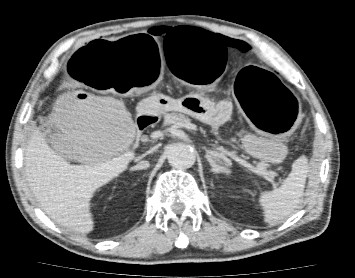
**Abdominal computed tomography on the fifth postoperative day.** A CT scan revealed severe colonic dilation. The diameter of the transverse colon was over 6cm. However, colonic wall thickening was absent.

**Figure 3:**
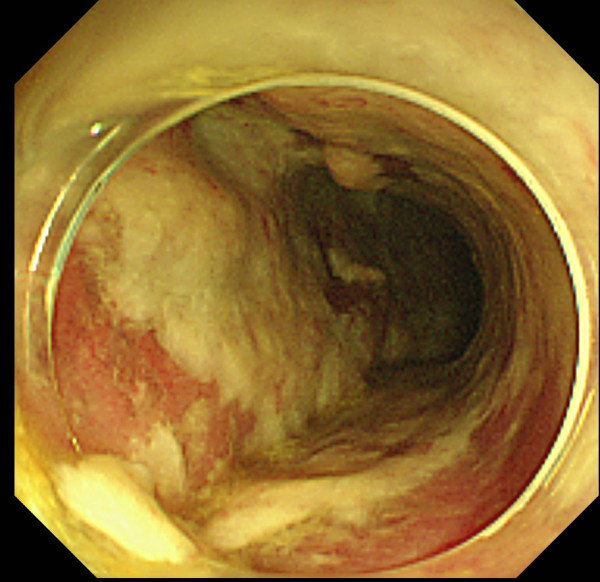
**Colonoscopy on the fifth postoperative day.** Colonoscopy demonstrated a colonic wall covered by thick pseudomembranes.

Nevertheless, in the early morning of the fifth postoperative day, his blood pressure continued to drop, thus requiring vasopressor treatment to maintain his blood pressure. Again, a CT was done, which indicated severe colonic dilation (Figure 2). Laboratory data were normal except for mild liver dysfunction (aspartate transaminase, 68mU/mL; alanine transaminase, 45mU/mL) and leukocytosis (9,460 cells/μL). Even though CT demonstrated severe dilatation of the colon, the thickening of the colonic wall that is the typical finding associated with fulminant pseudomembranous colitis was not evident. Emergent colonoscopy up to the transverse colon revealed pseudomembranes associated with severe inflammation (Figure 3). Pseudomembranous colitis was thus diagnosed, and oral vancomycin was administered. Until this time, no antibiotics had been given postoperatively. In our institute, the enzyme-linked immunosorbent assay (ELISA) for *C. difficile* toxin is not available on weekends (the fifth postoperative days was a Saturday).

The condition of our patient rapidly deteriorated so he was transferred to the intensive care unit at 10:30 a.m. After resuscitation, which included tracheal intubation and massive intravenous infusion along with multiple vasopressors, an emergent operation was done. The time interval between the drop of blood pressure and emergency surgery was 18 hours.

During the laparotomy, a small amount of bloody ascites was observed and the large intestine was severely dilated, part of which was necrotic. The serosa of the small intestine was slightly inflamed but there was no sign of ischemia. Total colectomy with creation of an end ileostomy was then performed, after which our patient was returned to the intensive care unit. However, he did not recover from the shock and died 26 hours after the surgery.

**Figure 4:**
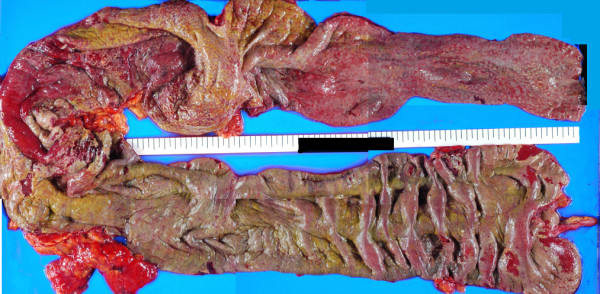
**Resected specimen.** Pseudomembranes were seen on the surface of the entire colon. The inflammation was more intense in the proximal aspect of the colon, which revealed significant edematous thickening associated with thick pseudomembranes

**Figure 5:**
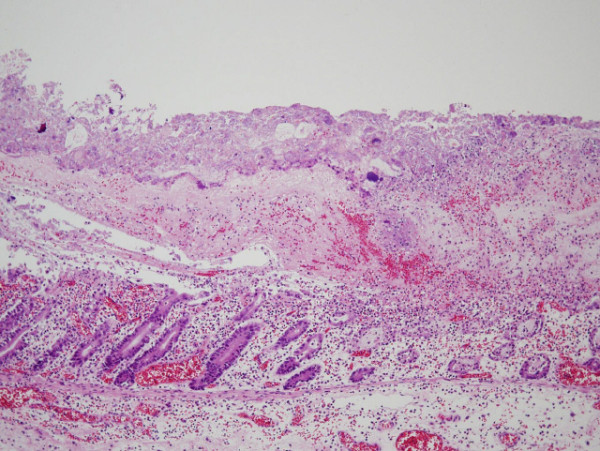
**Histopathological findings. **The histopathological examination revealed a necrotic exudate on the surface of the colonic mucosa. Severe inflammation was also seen, resulting in the disappearance of the upper part of the colonic crypts

A histopathological examination (Figures 4 and 5) revealed inflamed mucosa from the cecum to the rectum which was covered with a necrotic exudate, indicating pseudomembranous colitis. A postmortem autopsy was not carried out, because consent could not be obtained from the family.

## Discussion

Pseudomembranous colitis has been reportedly increasing, mainly attributed to wide usage of broad-spectrum antibiotics [2]. The clinical symptoms range from mild diarrhea to complicated systemic deterioration known as ‘fulminant colitis,’ which results in death or an illness severe enough that death would be likely without urgent colectomy [3]. Clinical manifestation of CDI is non-specific, including diarrhea, abdominal pain, fever, and leukocytosis. Disease-specific indicators include positive assay results for *C. difficile* toxin, colonic wall thickening revealed on CT and colonoscopic or histological identification of pseudomembranes [4].

According to the algorithm proposed by Jaber *et al*. [5], once CDI is suspected, the responsible antibiotic should be discontinued and treatment with oral vancomycin or metronidazole promptly started. Generally, the effect of medical treatment is evaluated 24 to 48 hours after its initiation; if it is considered to be ineffective, the indication for surgical treatment should be assessed. For patients requiring an operation, the mean time from onset of symptoms to diagnosis is 5.6 days. However, it is noteworthy that there are patients who do not respond to medical therapy and for whom the assessment period after the initiation of medical therapy merely delays the appropriate treatment for them, which is a total colectomy with creation of an ileostomy.

Patients who eventually undergo surgery should do so without wasting time with ineffective medical treatment and before the deterioration of their general condition occurs. But, this causes a dilemma, as earlier surgical intervention may result in an increased number of surgeries that could have been avoided by medical therapy. The generally accepted current algorithm for CDI reserves the surgical treatment for those with a deteriorated condition, such as hypotension, which necessitates vasopressor treatment, or shock, either at the initial presentation or during medical therapy [5]. However, it is apparent that the surgical outcome would be disastrous if the surgery was postponed until the deterioration of the general condition of the patient who was already sick [2,6]. In fact, surgical mortality is significantly higher in patients with shock compared to non-responders for medical treatment without shock [1].

It is well known that pseudomembranous colitis can develop in patients who have received only one dose of antibiotics [7,8]. Furthermore, although rare after minor operations, a surgical procedure is a risk factor of pseudomembranous colitis [9]. It is also reported that a substantial number of patients with fulminant CDI (36% to 75%) have a history of recent surgery [5]. Therefore, regardless of the severity of the surgery, all surgical patients should be managed as high-risk patients.

The clinical course of this patient was so rapid that cardiopulmonary function collapsed before the standard assessment period had barely begun. Our patient deteriorated into shock within a few hours of the start of medical treatment, which was initiated immediately after the diagnosis of pseudomembranous colitis by colonoscopy. Because of the possibility of the extremely rapid progression of this disease, as we experienced in this case, and the much higher mortality risk associated with surgery in patients with shock than without it, we propose that fast-track care, or an alternative to the conventional treatment algorithm, is desperately needed.

As this case has clearly revealed, the current algorithm for CDI does not facilitate the clinical management of fulminant pseudomembranous colitis, especially in critically severe cases. Considering the increasing morbidity of the pathology and recent outbreak of virulent strains, such as BI/NAP1/027 [1], we stress that the timing of a surgical consult should be earlier and the indication for surgery broader.

Early recognition of patients who are refractory to conventional medical therapy is important. Previous studies demonstrated significant predictors for fulminant colitis [1,4]. However, as far as postoperative patients in the surgical ward are concerned, clinical manifestation of CDI and predictors of fulminant colitis are not obviously distinguishable from symptoms seen in the uneventful postoperative course. In our case, for instance, the frequent bowel movement, which might have been the first symptom of CDI, was considered to have been the result of anal dysfunction after low anterior resection complicated with postoperative leakage. At present, it is practically extremely difficult to identify refractory cases before systemic deterioration develops.

ELISA-based assays for *C. difficile* toxin are rapid and specific and therefore have been widely used as a test for CDI [5]. However, the detection of this toxin in the stool does not automatically imply that CDI is severe or refractory to medical therapy. In order to assess the severity of colitis and necessity for emergent surgery, an evaluation of the colon is essential. Therefore, we consider that both enhanced abdominal CT and colonoscopy should be promptly performed once a patient’s postoperative course becomes aberrant from the expected one. We believe that they are the most reliable modalities for the evaluation of inflammation of the colon. If colonoscopy reveals confluent, not spotty, pseudomembranes in considerable parts of the large intestine, or CT demonstrates severe wall thickening or dilatation of the colon, emergent surgery might be the treatment of choice for survival.

## Conclusions

We presented a case with fatal fulminant pseudomembranous colitis. The clinical course rapidly progressed beyond the scope of the conventional management algorithm. It is important to recognize that, although rare, there is a type of extremely aggressive pseudomembranous colitis in which the waiting period for medical treatment might be lethal and, therefore, immediate surgery should be performed. We believe that colonoscopy and CT are helpful to decide the necessity of emergent surgical treatment without delay.

## Consent

Written informed consent was obtained from the patient’s family for publication of this case report and any accompanying images. A copy of the written consent is available for review by the Editor-in-Chief of this journal.

## Competing interests

The authors declare that they have no competing interests.

## Authors’ contributions

IA was the main contributor to the preparation of the rough draft, and to the analysis and interpretation of the data under the supervision of YJK. JS contributed to the acquisition of data. FK critically revised the manuscript. All authors read and approved the final manuscript.
